# At-Risk Newborns: Overlooked in Expansion From Essential Newborn Care to Small and Sick Newborn Care in Low- and Middle-Income Countries

**DOI:** 10.9745/GHSP-D-22-00099

**Published:** 2023-02-28

**Authors:** Indira Narayanan, James A. Litch, Ganga L. Srinivas, Kwabena Onwona-Agyeman, Alhassan Abdul-Mumin, Jayashree Ramasethu

**Affiliations:** a Georgetown University Medical Center, Washington, DC, USA.; b Global Alliance to Prevent Prematurity and Stillbirth, Lynnwood, WA, USA.; c Case Western Reserve University, Cleveland Medical Center/Rainbow Babies and Children’s Hospital, Cleveland, OH, USA.; d Division of Neonatal Care Unit, Department of Pediatrics, Greater Accra Regional Hospital, Accra, Ghana.; e Department of Pediatrics and Child Health, School of Medicine, University for Development Studies; Department of Pediatrics and Child Health, Tamale Teaching Hospital, Tamale, Ghana.; f MedStar Georgetown University Hospital, Washington, DC, USA.

## Abstract

We propose adding the category of “at-risk” newborns for babies who are at increased risk of morbidity and/or mortality but do not require special or intensive care or monitoring to promote a 3-tiered newborn care approach in hospitals.

## Key Messages

- In global health, the initial focus on essential newborn care for all now includes adding specialized care for small and sick newborns.
- In many low- and middle-income countries (LMICs), this has resulted in transferring relatively stable newborns with risk factors who require observation and minimal support to special/intensive care units that are already overcrowded and understaffed.
- To prevent needless separation from mothers and overcrowding in neonatal units, we propose an “at-risk” category of newborns and suggest feasible, more cost-effective options to manage these newborns at health care facilities in LMICs.

## AN EVOLVING APPROACH TO NEWBORN CARE

Between 1990 and 2017, the global under-5 mortality rate declined by 58% and the neonatal mortality rate by 51%,^1^ but both measures remain well above the Sustainable Development Goal targets for 2030.^2^ In many low- and middle-income countries (LMICs), major challenges persist, hindering the achievement of Sustainable Development Goal 3.2, in particular, of reducing preventable deaths of newborns to at least 12 deaths per 1,000 live births by the year 2030.^2^

Newborn care in LMICs has varied based on factors such as the low availability of funds and the competing priorities of country governments, various stakeholders, and the private sector. Newborn care was initially viewed as a high-tech intervention, and resources were consequently focused on level III neonatal intensive care units (NICUs) and level II special neonatal care units (SNCUs) in hospitals, even when home deliveries were common. Community-based newborn care was positioned under the larger umbrella of maternal and child health, with the newborn components often slipping between the 2 pillars of maternal health and child health.

Over the last 2 decades, a more coordinated attempt to address newborn care has been implemented in LMICs through programs such as Basic Support for Institutionalization of Child Survival (BASICS),^3^ Maternal and Newborn Health Program,^4^ Saving Newborn Lives,^5^ Every Preemie–SCALE (Scaling, Catalyzing, Advocating, Learning, Evidence-Driven),^6^ and Every Newborn Action Plan.^7^ Unlike maternal health that focused primarily on skilled birth attendance at deliveries, newborn care programs began with community-based care, centering around the concept of essential newborn care (ENC) for all babies. This was to a considerable extent influenced by the seminal study on home-based care of the newborn by Bang et al. in 1999.^8^ ENC comprised (1) basic care, including clean delivery practices, temperature maintenance with immediate drying, skin-to-skin contact, early initiation of breastfeeding, clean cord care, eye care, basic resuscitation; and (2) identification of and referral for common problems/danger signs.^9^ ENC later expanded to include other components, such as kangaroo mother care and the detection and treatment of infections. The content of ENC varies in different countries and regions, with basic clinical components at the community level and additional elements, including investigations, in health facilities. ENC has become a fundamental requirement, and nearly all national health authorities have adopted it as their standard of basic care.^10^^,^^11^

When prematurity was identified as the leading cause of under-5 mortality in 2015, the need to provide appropriate facility-based care for the small and sick newborns was renewed,^12^ as was the need to provide 3-tiered care, appropriate to resources in the community, at first and second level facilities, and finally referral/tertiary level hospitals.^13^

## THE NEED FOR AN INTERMEDIATE NEWBORN CATEGORY

The label “small and sick newborn” has served well for early identification of infants who require a higher acuity of monitoring and care, including intensive interventions, and for differentiation from the “well newborn.” This clear demarcation allows infants who fall outside the small and sick category to remain with their mothers to bond and establish breastfeeding with minimal concerns for deterioration. The approach addresses hospital-based care, as many primary health care facilities/centers below district-level hospitals lack the resources for providing more than basic care and can keep mother-baby dyads only for a few hours or at most a day and thus refer babies with potential or actual problems to district, regional, or tertiary hospitals.^14^

In several LMICs, notably in sub-Saharan Africa, mothers and their well babies are provided care in postnatal wards only by midwives whose primary expertise and focus is maternal care. Training in the care of newborns is minimal, and newborn care does not always extend to periodic monitoring. Newborns in the postnatal wards are also not routinely examined by the neonatal unit physicians because regular physician/pediatrician rounds do not take place in such postnatal wards, and doctors assess a baby only when referred by the midwife (Ashura Bakari, personal communication, 2021). A study of facility-based newborn care in India noted concerns with the quality of care with respect to rounding by midwives and availability of attending physicians.^15^ In the global shift from ENC provided at home/community and at peripheral centers by midwives to care of small and sick newborns at the SNCU/NICU in hospitals, insufficient attention has been directed to the quality of care in upper-level hospital postnatal wards. This relates, in particular, to suitable training of midwives and supply of relevant basic resources, with, ideally, routine physician rounding to identify and manage newborns with higher acuity problems. As a result, at birth, newborns with risk factors requiring some additional monitoring and even low-acuity interventions are grouped with the small and sick newborns and, although clinically stable, are admitted to the SNCU/NICU. In single-center reports of NICUs in Jordan and Nepal, the authors noted a substantial percentage of admissions for maternal premature rupture of membranes of more than 18 hours (Jordan 21%; Nepal 12%) and for physiologic jaundice (Jordan 10.7%; Nepal 7%).^16^^,^^17^

This dichotomous newborn care model of intensive/special care versus minimal to no physician-level care in many facilities in LMICs, particularly in sub-Saharan Africa, may ensure better monitoring and avoid deterioration going unrecognized in minimally staffed postnatal wards. In our opinion, this also has the potential for overmedicalization of care of babies who require only some additional observation and minimal extra care and can add considerably to the workload of the already crowded and understaffed advanced neonatal units. A study of care in 16 referral hospitals in Uganda, Indonesia, and India described the nurse-to-patient ratio in some hospitals in LMICs can be as high as 1:15 during the day and 1:30 at night, with some units having more than 1 baby sharing a cot, radiant warmer, or incubator.^18^ Transfers to the neonatal unit thus have the potential to expose babies to hospital-acquired infections, especially in understaffed, crowded units. This practice also needlessly separates newborns from their families, with adverse impacts on both babies and mothers, including on establishing breastfeeding and bonding in this vulnerable subgroup.

This dichotomous newborn care model has the potential for overmedicalization of care of babies who require only some additional observation and minimal extra care.

## DEFINING THE AT-RISK NEWBORN

We propose the term “at-risk newborn” to describe a neonate with an increased risk of morbidity and/or mortality but who is currently maintaining homeostasis and does not require a special or intensive care level of monitoring or medical intervention. Such at-risk babies require close monitoring with simple technology for a period of time, which can be provided by a few additional health caregivers with some additional newborn care training above ENC but not to the level of nurses in level II or III neonatal units. This allows these newborns to continue to adapt well to extrauterine life without separation from their mothers and provides for the timely recognition of conditions that require transfer to the SNCU/NICU. Some of these at-risk babies may also merit subsequent closer evaluation at home during follow-up.

Although there is an existing 3-tiered approach of basic/essential newborn care at home or a primary care facility, with transfer to level II care and level III care at a different facility when needed, the system used only consists of 2 newborn categories, “well” and “small and sick.”^12^^,^^13^ Our concept of “at-risk” babies focuses on the delivery of care to this special group of babies in individual hospitals that already have either level II or level III/IV care. In both the latter types of care, there often appears to be a 2-tiered approach of either essential newborn care or special/intensive care. Our opinion is that, especially for care strategies for babies delivered in higher-level facilities, identifying and adding a group of babies with a specific label of the “at-risk” newborn will help promote a 3-tiered approach in each hospital of (1) basic care, (2) care of the at-risk babies, and (3) special/intensive care. This proposed framework not only formalizes the existing tiering of newborn care between primary, district, and tertiary care facilities but also clarifies and systematizes strategies for care delivery within the latter 2 levels of facilities, thus promoting nonseparation from mothers for more babies.

Identifying and adding a group of babies with a label of “at-risk” newborns will help promote a 3-tiered approach in each hospital.

The Box lists examples of at-risk newborns who appear well at birth but are at higher risk for developing problems than well babies with no identifiable risk factors. Some small newborns who currently fit into the small and sick newborn category may also not immediately need the interventions traditionally available in level II or level III neonatal care units and may be better grouped as at-risk newborns. Monitoring of these at-risk newborns with early identification and timely management of problems is necessary for better outcomes, and it is important that the resources and training are appropriate.^19^ However, this at-risk group has not always been clearly delineated in national action plans. The Ghana Health Service National Newborn Health Strategy and Action Plan is one that does at least identify this at-risk group of babies as a specific entity that needs to be addressed, although it does not outline separate care strategies.^20^

BOX **Characteristics of At-Risk Newborns Who Can Be Roomed-In With Mothers and/or Surrogates**

Newborns who merit increased monitoring and additional support not equivalent to special or intensive care can room with mothers and/or other family members such as grandmothers, fathers, and/or appropriate close friends if the mothers are critically ill, in isolation, or dead. These include babies with the following characteristics.

- Birthweight ≥1800 gm
- Gestational age ≥34 weeks
- Large for gestational age and having a diabetic mother
- Asymptomatic but at risk for sepsis, including those receiving antibiotics
- Meconium-stained amniotic fluid but baby otherwise stable
- Birth asphyxia with rapid revival (Apgar score >7 at 5 minutes)
- Mild to moderate jaundice needing monitoring, with or without phototherapy
- Blood group incompatibilities
- Syphilis/HIV-exposed
- Receiving feeding support but not requiring tube feeds
- Temperature instability responding to skin-to-skin contact
- Without apparent problems but with maternal critical illness/mortality

Acuity different from any suggested in this list may be determined by the human and material resources available to manage at-risk newborns at each facility outside the special neonatal care units/neonatal intensive care units. This includes gestational age and birthweight limits.

It is important to differentiate this categorization of an at-risk newborn from the at-risk approach related to maternal health.^21^ The aim is not to have a targeted at-risk approach to only identify babies with risk factors and provide necessary care but to promote (1) essential care for all newborns, (2) additional monitoring and support in an appropriate manner for more vulnerable at-risk babies, and (3) special/level II and intensive/level III/IV care reserved for overtly unstable newborns requiring advanced care. Central to this concept is the promotion of nonseparation of the mother and baby, ideally for all categories, but at a minimum for the well newborns and the at-risk group.

Although data from LMICs are lacking, in high-income countries (HICs), late preterm infants constitute about 60% of all preterm babies and 4%–7% of all live births.^22^^,^^23^ These babies represent a prime example of an at-risk category with increased risk of morbidity and mortality as compared with full-term newborns.^24^ Too early discharge may result in rehospitalization of these babies with problems such as poor feeding, hypothermia, hypoglycemia, and significant jaundice.^25^ At-risk categorization could facilitate the extension of closer monitoring of late preterm infants for the few days required to ensure that homeostasis is achieved before discharge, while maintaining the mother-baby dyad. Other at-risk newborns will similarly need extra monitoring to identify potential clinical problem(s) and institute the necessary interventions within the capacity of the nonintensive setting to treat and halt progression. This can also facilitate early identification of deterioration to permit timely transfer to the higher level of care in the SNCU/NICU.

## APPROACHES FOR NEWBORN CARE

The Table lists suggested levels of care for the 3 categories of newborns. Besides the necessary infrastructure, human resources, and supplies, the family-centered care approach would be an ideal overarching requirement for all options.^27^

**TABLE.** Recommendations for Level of Facility Care for the 3 Categories of Newborns

	**Well Newborns**	**At-Risk Newborns**	**Small and Sick Newborns**
Location/placement	Postnatal ward/postpartum unit (wards or single rooms)	Roomed-in with mothers in a designated areaAttached to/in the postnatal ward/postpartum unitAttached to SNCU/NICU^a^Kangaroo mother care units	Special neonatal care unit/neonatal intensive care unitMother-baby unit ^b^
Health care provider	Midwives/nurses well trained in the basic care of both the mother and baby, preparation for discharge, and monitoring for and identification of danger signsIdeally and where feasible, coupled with daily rounding by trained physician assistants, nurse practitioners, physicians, pediatricians, and/or neonatologists	Midwives/nurses trained additionally in the monitoring and care of the at-risk babiesDaily rounding by trained physician assistants, physicians, pediatricians, and/or neonatologists with additional visits as required (more frequent than for well babies)	Nurses trained to provide level II, III, and IV care^c^Pediatricians, neonatologists, and/or midlevel providers trained to provide level II, III, and IV care

a Typically, special neonatal care units/neonatal intensive care units do not support rooming-in care for the postpartum mother.

b Neonatal units, often level II, where small and sick newborns are kept with their mothers, separate from the general postnatal wards/postpartum units (as in some sub-Saharan countries).

c Level 1 care: resuscitation at delivery and postnatal care to stable term and late preterm infants (35–<37 weeks of gestation) and stabilization and transport to all others; level II care: >32 weeks of gestation and >1500 g, or moderately ill with conditions expected to resolve rapidly; level III/IV: provide sustained life support and comprehensive care including access to pediatric subspecialists (American Academy of Pediatrics Committee of Fetus and Newborn).^26^

Postnatal/postpartum units may exist as wards or single rooms, the latter being more common in HICs and in some private facilities in LMICs. Where these units are used for at-risk newborns, a special demarcated area or room, preferably located near the nursing station, would permit easy identification for providing the extra required nursing care and additional visits by the physicians, as well as ensure that such mother-baby dyads are not inadvertently discharged too early. Ideally, the inclusion of nursing staff with additional training in this section would be very beneficial, though competence to the extent of the SNCU/NICU nursing staff may not be necessary. Equal emphasis on the care of the mother and the baby by trained staff, including physicians and nurses, should be promoted in these units.^28^ Also, as noted in the Table, the adapted postnatal mother-baby unit may be linked with the postnatal ward^29^ (Sushma Nangia, personal communication) or with the SNCU/NICU.^30^ In their review of the tiered approach in India, Neogi et al. describe an approach of 3 levels of newborn care (though the at-risk group is not named) that includes observation in a newborn stabilization unit for vulnerable babies that are in need of transfer to a special care unit.^15^ The at-risk approach we describe has some similarities to the newborn stabilization units in India. However, these units are usually in lower-level centers, where babies brought from home are stabilized and kept until they are ready for discharge or to be transferred to a higher-level hospital for more advanced care. In contrast, the at-risk babies we focus on in this article are those babies who are delivered in hospitals that already have the level II, III, or IV neonatal unit.

In some district-level hospital SNCUs in sub-Saharan African countries, provision of level II care may also be implemented in a mother-baby unit. These units are distinct from postnatal/postpartum wards in both staffing and admission criteria (Ashura Bakari, personal communication). There is now evidence to suggest that the ideal option for all categories of care may be the mother-baby unit with no separation, using kangaroo mother care and family-centered care wherever feasible for low birthweight infants, beginning at birth.^27^^,^^31^ However, this requires careful planning, appropriate infrastructure, beds, equipment, supplies, and suitably trained, motivated administrative staff and health care providers, as well as, most importantly, a shift in the culture of care.

### Other Considerations of the At-Risk Newborns

There is a shortage of human resources and supplies in many health care facilities in LMICs, and any of the previously mentioned options for the care of the at-risk newborn group will require space, equipment, and extra staff.^32^ As noted, although these staff will require more training than those managing babies in the conventional postnatal ward, the additional skills needed do not approach the complexity of training required for level II/SNCU and level III/NICU care.

Naming an at-risk newborn group will provide clarity to all health systems to pursue a differentiated 3-tiered approach (well care vs. low acuity monitoring and intervention vs. special/intensive care). This will also help to reduce the census in crowded neonatal units, reducing the demand for highly skilled staff who will be better able to focus on babies requiring special/intensive care. A further benefit would be in prioritizing outpatient care. Neogi et al. describe a pilot intervention of follow-up of small and sick babies through a mobile phone–enabled tracking system at more frequent intervals than well infants, with plans to expand to other primary care units.^15^ This approach to follow-up could also include at-risk babies, and such categorization would facilitate closer follow-up with greater intensity than for the low-risk babies but somewhat lesser intensity than that required for small and sick newborns.

Care processes can also incorporate early warning scales that have been shown to help staff identify deterioration early in adults^33^ as well as in children.^34^ Attempts have been made to develop neonatal early warning scoring scales for use by midwives and nurses in postnatal wards and referral health centers to more objectively recognize clinical deterioration early and initiate prompt transfer to higher-level care.^35^

In HICs as well, NICUs are increasingly taking on the care of these at-risk babies. In recent years, more than 95% of births and 80% of admissions involve more mature babies above 34 weeks of gestational age that spend very few days in the NICU.^36^ Questions are being raised even in HICs as to whether newborns in the lower range of acuity can be more appropriately cared for in a setting other than an intensive care neonatal unit.^37^

The exact classification and care for at-risk newborns may vary in different countries and facilities. Ultimately, while standards can be recommended, each baby and mother should be evaluated on an individual basis in the various facilities to match the care required to available resources. However, changing systems will allow us to practice good stewardship in the management of the at-risk group, reducing strain on the limited resources in LMICs essential for the small and sick newborns and avoiding unnecessary separation of the baby and mother.

Ultimately, while standards can be recommended, each baby and mother should be evaluated on an individual basis in the various facilities to match the care required to available resources.

## CONCLUSION

Newborn care in LMICs has transitioned rather abruptly from ENC for all newborns to a focus on special/intensive care for the small and sick baby without considering an intermediate group of at-risk newborns. Expanding current recommendations to include and define this at-risk group can help delineate the structure, processes, and personnel that are appropriate for each subgroup, enabling better stewardship of scarce resources and avoidance of potential overmedicalization. We feel that this 3-tiered care approach could also be considered for inclusion in the new Implementation Tool for Small and Sick Newborn Care recently launched on World Prematurity Day 2021.^38^ This may actually help improve health outcomes, as the increased awareness of the importance of monitoring and caring of at-risk babies in an appropriate manner could provide a better safety net of care for all babies, in addition to preventing overcrowding and avoiding hospital-acquired infections in the SNCU/NICU.

Incorporating the concept of the at-risk newborn into the existing paradigm of well newborn and small and sick newborn can encourage countries and facilities to review their policies and target their approach in a suitable manner. This can promote provision of the appropriate level of care for each of the 3 categories and also support care of the newborn and mother as a dyad within the context of kangaroo mother care and family-centered care, with implications for the intensity and cadence of monitoring at home after discharge.

## Notes

**Peer Reviewed**

**First published online:** January 12, 2023.
